# ﻿Revisionary notes on the genus *Aulacocentrum* Brues (Hymenoptera, Braconidae, Macrocentrinae) from Vietnam

**DOI:** 10.3897/zookeys.1197.116092

**Published:** 2024-04-03

**Authors:** Thi Nhi Pham, Khuat Dang Long, Cornelis van Achterberg, Thi Quynh Nga Cao, Van Phu Pham, Thi Hoa Dang

**Affiliations:** 1 Institute of Ecology & Biological Resources (IEBR), Vietnam Academy of Science & Technology (VAST), 18 Hoang Quoc Viet Road, Cau Giay, Ha Noi, Vietnam Institute of Ecology & Biological Resources (IEBR), Vietnam Academy of Science & Technology (VAST) Ha Noi Vietnam; 2 Graduate University of Science & Technology, Vietnam Academy of Science & Technology (VAST), 18 Hoang Quoc Viet Road, Cau Giay, Ha Noi, Vietnam Graduate University of Science & Technology Ha Noi Vietnam; 3 Naturalis Biodiversity Centre, Postbus 9517, 2300 RA Leiden, Netherlands Naturalis Biodiversity Centre Leiden Netherlands

**Keywords:** Australasian, Eastern Palaearctic, Ichneumonoidea, new record, new species, Oriental, parasitoid

## Abstract

This paper contains descriptions and illustrations of five new species of the genus *Aulacocentrum* Brues, 1922, from Vietnam, viz. *Aulacocentrumassitum* Long & Pham, **sp. nov.**; *A.glabrum* Long, **sp. nov.**; *A.imparum* Long & van Achterberg, **sp. nov.**; *A.intermedium* Long & van Achterberg, **sp. nov.**; and *A.simulatum* Long, **sp. nov.** Additionally, *Aulacocentrumseticella* van Achterberg & He is newly recorded for Vietnam’s braconid fauna. A checklist and a key to the Oriental and East Palaearctic *Aulacocentrum* species is provided and the in-country distribution of the Vietnamese species is given.

## ﻿Introduction

Macrocentrinae Foerster, 1863, is a relatively small subfamily of Braconidae Nees, 1811, comprising eight recognised genera. *Aulacocentrum* Brues, 1922 is a rather small genus distributed in the Old World tropics and southern part of the East Palaearctic region. Up to now, *Aulacocentrum* comprises nine valid species, of which four species are known from the Oriental region (but the position of one is uncertain) and one species additionally from the East Palaearctic region.

*Aulacocentrum* differs from other macrocentrine genera by having the first metasomal tergite with fine transverse striation and vein SC+R1 of hind wing strongly bent basally, both absent in *A.glabrum* sp. nov., but has typical narrowed marginal cell of hind wing (Fig. 4F). A detailed diagnosis of the genus *Aulacocentrum* was given by van Achterberg (1993b) and He and van Achterberg (1994). Previous records indicate that *Aulacocentrum* species are mainly endoparasitoids of pyralid larvae (Lepidoptera) (He and van Achterberg 1994; Yu et al. 2016). In this paper seven species of the genus are reported from Vietnam, of which five are new to science and one species is newly recorded for the fauna of Vietnam.

The species of the genus *Aulacocentrum* have been reported as a larval parasitoid of economically important pyralid moths; *Aulacocentrumphilippinense* (Ashmead), a widespread species, was reported from *Botyodesasialis* Guenée; *Chilosuppressalis* (Walker); *Cnaphalocrocismedinalis* (Guenée); *Crocidolomiapavonana* (Fabricius); *Diaphaniapyloalis* (Walker); *Marucavitrata* (Fabricius); *Palpitanigropunctalis* (Bremer); and *Spoladearecurvalis* (Fabricius) (Yu et al. 2016). In Vietnam, *A.philippinense* was previously reported as endoparasitoid reared from the rice leaf roller *Cnaphalocrocismedinalis* (Long and Belokobylskij 2003). In China, *A.confusum* He and van Achterberg was reported as parasitoid of several pyralid and crambid moths, such as *Ostriniafurnacalis* (Guenée), and *Diaphaniapyloalis*; and *A.seticella* was recorded as a parasitoid of *Pachyzancla* sp. (He and van Achterberg 1994).

## ﻿Materials and methods

All *Aulacocentrum* specimens, including holotypes and paratypes, are deposited in the Institute of Ecology & Biological Resources (**IEBR**) at Ha Noi, Vietnam. The collecting was by light traps in open spaces in the forest, but *A.seticella* was collected in Malaise traps.

We used an Olympus® SZ61 binocular microscope for this study; specimens were photographed by KDL using a Sony® 6000 digital camera attached to a Nikon® SMZ 800N binocular microscope and the figures were processed with Helicon Focus^®^8 stacking software and Adobe Photoshop CS5 to adjust the size and background. The distribution map for the two new species of *Aulacocentrum* was made using Paraview (https://paraview.org).

For terminology used in this paper, see van Achterberg (1993a), sculpture terms are based on Harris (1979), and vein terminology follows the modified Comstock-Needham system (van Achterberg 1993a). For a key to genera of Macrocentrinae in the Palaearctic region, see van Achterberg (1993b). For additional references and data, see Yu et al. (2016). Inside Vietnam, the distribution of the species follows the order of areas and provinces from north to south, and outside Vietnam, distribution of species follows an alphabetical order.

Abbreviations used in this paper are as follows:

**POL** minimum postocellar line;

**OOL** minimum ocular-ocellar line;

**OD** maximum diameter of posterior ocellus;

“**Macr.**+**number**” code number indexing for Macrocentrinae specimens in the collection at IEBR;

**MT** Malaise trap;

**NE** Northeastern;

**S** South;

**HTHCT** Department of Insect Systematics at IEBR;

**IEBR** Institute of Ecology & Biological Resources, Vietnam Academy of Science and Technology, Ha Noi, Vietnam.

## ﻿Results

### ﻿Taxonomy

**Class Hexapoda Blainville, 1816**

**Order Hymenoptera Linnaeus, 1758**

**Superfamily Ichneumonoidea Latreille, 1802**

**Family Braconidae Latreille, 1829**

**Subfamily Macrocentrinae Foerster, 1863**

#### ﻿Genus Aulacocentrum

Taxon classificationAnimaliaHymenopteraBraconidae

Brues, 1922

030CDEEB-A95B-5160-BAAA-235DF1D4ABEF

##### Type species.

*Aulacocentrumpedicellatum* Brues, 1922 (examined by CvA).

### ﻿Checklist and distribution of Oriental and East Palaearctic *Aulacocentrum* species

*Aulacocentrumassitum* Long & Pham, sp. nov. Oriental/Vietnam.

*Aulacocentrumconfusum* He & van Achterberg, 1994. Eastern Palaearctic and Oriental/China.

*Aulacocentrumglabrum* Long, sp. nov. Oriental/Vietnam.

*Aulacocentrumimparum* Long & van Achterberg, sp. nov. Oriental/Vietnam.

*Aulacocentrumintermedium* Long & van Achterberg, sp. nov. Oriental/Vietnam.

*Aulacocentrumlongitergiae* Sharma, 1978. Oriental/India.

*Aulacocentrumnigrum* Ku & Park, 1997. Eastern Palaearctic/Korea.

*Aulacocentrumphilippinense* (Ashmead, 1904). Australasian/Indonesia-South Moluccas; Eastern Palaearctic/China, Japan, Korea; Oriental/India, Indonesia, Malaysia, Philippines, Vietnam.

*Aulacocentrumseticella* van Achterberg & He, 1994. Eastern Palaearctic/China, Japan, Korea; Oriental/China, India, Indonesia, Malaysia, Singapore, Vietnam.

*Aulacocentrumsimulatum* Long, sp. nov. Oriental/Vietnam.

### ﻿Key to Oriental and East Palaearctic *Aulacocentrum* species

**Table:** 

1	Female	**2**
–	Male (as far as known)	**10**
2	Vein SR of hind wing strongly bent basally, and near to or almost touching anterior wing margin at constriction (Figs 2I, 13D); first metasomal tergite flat basally (Fig. 2F)	**3**
–	Vein SR of hind wing curved to moderately bent basally, remaining distinctly removed from anterior wing margin (Figs 4F, 6J, 8H, 10J); first metasomal tergite with basal depression (Figs 4G, 6F, 8I, 10K, 12I), rarely with shallow depression basally (*A.philippinense*)	**4**
3	Maxillary palp 2.2× height of head; fore wing vein 1-CU1 0.4× as long as vein cu-a (Fig. 2H); hind wing marginal cell parallel-sided apically (Fig. 2I); maximum length of second submarginal cell of fore wing 2.4× its apical width; vein 1r-m of hind wing 1.1× as long as vein 1-M and vein 1-M 0.6× as long as vein cu-a; notauli separated posteriorly by longitudinal carina-like rugosity (Fig. 2D); first metasomal tergite smooth basally; propleuron dark brown; hind coxa brown apically; first metasomal tergite black apically (Fig. 2F)	***A.assitum* Long & Pham, sp. nov.**
–	Maxillary palp 1.6–1.7× height of head; fore wing vein 1-CU1 0.8× vein cu-a; hind wing marginal cell widened apically (Fig. 13D); maximum length of second submarginal cell of fore wing 3.0× its apical width; vein 1r-m of hind wing 0.9× as long as 1-M and 1-M 0.9× vein cu-a; notauli fused posteriorly into transverse rugulose area (Fig. 13B) or ending in a long carina; first metasomal tergite with sparse, convergent striations basally; propleuron yellow (Fig. 13C); hind coxa and first metasomal tergite yellow	***A.seticella* van Achterberg & He**
4	Ocelli large, OOL = 0.5–0.6× OD (Figs 4A, 12A); first metasomal tergite with deep medio-basal depression (Figs 4G, 12I)	**5**
–	Ocelli small to medium-sized, OOL = 1.0–1.1× OD (Figs 6A, 10A; see van Achterberg 1993b: fig. 3); first metasomal tergite flat or with shallow medio-basal depression (Figs 6F, 10K)	**6**
5	Vein SC+R1 of hind wing curved basally; basal cell apically and marginal cell basally of hind wing largely glabrous (Fig. 4F); marginal cell of hind wing distinctly narrowed medially and widened apically (Fig. 4F); first metasomal tergite with striation subapically (Fig. 4G)	***A.glabrum* Long, sp. nov.**
–	Vein SC+R1 of hind wing distinctly bent basally; basal cell apically and marginal cell basally of hind wing setose (Fig. 12G); marginal cell of hind wing weakly narrowed medially and subparallel-sided apically (Fig. 12G); first metasomal tergite with transverse or curved transverse striation subapically (Fig. 12I)	***A.simulatum* Long, sp. nov.**
6	Antennal flagellum without median pale coloured (ivory) flagellomeres; all coxae and metasomal tergites mainly black; vein 1r-m of hind wing 0.6× as long as vein 1-M; scapus black ventrally; [first tergite ~ 3.7× its apical width]	***A.nigrum* Ku & Park**
–	Antennal flagellum with median pale coloured (ivory) flagellomeres; fore and middle coxae yellow and hind coxa reddish yellow; basal 1/3 of first tergite and basal 2/3 of third tergite pale yellow (Figs 6F, 10K); vein 1r-m of hind wing 0.8–0.9× as long as vein 1-M; scapus whitish yellow or dark brown ventrally (Figs 6B, 10B)	**7**
7	Clypeus basally less convex (He and van Achterberg 1994: figs 38, 39); malar space comparatively short (He and van Achterberg 1994: fig. 32); scapus dark brown, similarly coloured as first flagellomere or distinctly darker; hind trochantellus with 5–10 teeth, usually in two or three rows (He and van Achterberg 1994: figs 35, 42)	***A.confusum* van Achterberg & He**
–	Clypeus basally more convex (Figs 6B, 10B, 15B; He and van Achterberg 1994: figs 25, 26); malar space longer (Figs 6B, 10B, C; He and van Achterberg 1994: fig. 26); scapus at least partly ivory or pale yellowish ventrally, much paler than dark brown or blackish first flagellomere; teeth on hind trochantellus variable	**8**
8	First metasomal tergite basally flat or near so (see Fig. 5 in van Achterberg 1993b); laterope more or less differentiated from glymma; second tergite distinctly constricted medio-laterally; hind coxa largely smooth with some transverse striae apically; hind femur mostly yellowish brown apically and yellow basally (but hind femur dark brown in males)	***A.philippinense* (Ashmead)**
–	First metasomal tergite with basal depression (Figs 6F, 10K); laterope large, merged into deep groove posteriorly (Figs 6H, 10G); second tergite slightly constricted medio-laterally (Figs 6F, 10K); hind coxa punctate with transverse oblique striae apically (Fig. 6G); hind femur mostly dark to blackish brown and brownish yellow at extreme base	**9**
9	First metasomal tergite with dorsal carinae (Fig. 6F); vein 1-SR+M of fore wing angularly bent medially (Fig. 6I); vein SC+R1 of hind wing less bent (Fig. 6J); vein 2-SC+R of hind wing quadrate (Fig. 6J); length of hind femur 8.4× its maximum width; hind coxa remotely punctate dorsally, with oblique striae dorso-apically (Fig. 6G); mesopleuron, metapleuron and propodeum black (Fig. 6C)	***A.imparum* Long & van Achterberg, sp. nov.**
–	First metasomal tergite without dorsal carinae (Fig. 10K); vein 1-SR+M of fore wing evenly curved medially (Fig. 10H); vein SC+R1 of hind wing more bent (Fig. 10J); vein 2-SC+R of hind wing longitudinal (Fig. 10J); length of hind femur 9.2× its maximum width; hind coxa densely punctate dorsally, with transverse striae dorso-apically (Fig. 10I); mesopleuron dark brown, metapleuron entirely, propodeum basally and laterally pale yellow (Fig. 10F)	***A.intermedium* Long & van Achterberg, sp. nov.**
10	Antennal flagellum unicoloured, without median pale flagellomeres; head and hind tibia reddish brown; length of first tergite 6.0× its apical width [marginal cell of hind wing strongly constricted subbasally; female unknown; India]	***A.longitergiae* Sharma**
–	Antennal flagellum bicoloured, with median pale flagellomeres (Figs 7, 14); head entirely black (Figs 8A, 15A); hind tibia mostly blackish brown to black; length of first tergite 3.8–4.3× apical width	**11**
11	Clypeus less convex; malar space rather short (Fig. 8C); first metasomal tergite parallel-sided, with rather deep basal depression (Fig. 8I); vein 1-SR+M of fore wing angularly bent medially (Fig. 8F); vein 1r-m of hind wing 0.7× 1-M; vein 2-SC+R of hind wing quadrate (Fig. 8H); hind coxa rather short, distinctly depressed dorso-apically, sparsely punctate dorsally and smooth apically (Fig. 8G); body length 7.8 mm	***A.imparum* Long & van Achterberg, sp. nov.**
–	Clypeus more convex; malar space longer (Fig. 15C); first metasomal tergite gradually widened posteriorly, with shallow medio-basal depression (Fig. 15H); vein 1-SR+M of fore wing evenly curved medially (Fig. 15I); vein 1r-m of hind wing 0.9× 1-M; vein 2-SC+R longitudinal (Fig. 15J); hind coxa elongate, sparsely punctate dorsally, weakly depressed dorso-apically and with some fine transverse striae apically (Fig. 15G); body length 7.5–9.0 mm	***A.philippinense* (Ashmead)**

The type species, *Aulacocentrumpedicellatum* Brues (described from Fiji but also known from the Australian region) is not included in the key because it has never been found in the Oriental region. *Aulacocentrumpedicellatum* Brues can be separated by the following characters: widened subbasal part of marginal cell of hind wing largely glabrous, rarely only medially glabrous (see He and van Achterberg 1994: figs 24, 37); length of first tergite 5.0–8.0× its apical width; apical 1/2 of wing membrane infuscate; mesosoma dark reddish brown; length of body usually more than 11.0 mm. The position of *A.longitergiae* Sharma, 1978 is uncertain because the holotype is unavailable for study. According to the author, the holotype was largely lost during handling (Sharma pers. comm. to CvA July 1985). *Aulacocentrumlongitergiae* Sharma is provisionally included and may be distinguished as follows: widened subbasal part of marginal cell of hind wing completely setose; length of first tergite 6.0× its apical width; apical 1/2 of wing membrane hyaline; mesosoma reddish brown or infuscate; length of body less than 11.0 mm; flagellum unicoloured (in male, female unknown), without pale flagellomeres; head and hind tibia reddish brown (Sharma 1978; He and van Achterberg 1994).

For the redescription of *A.philippinense* (Ashmead), see van Achterberg (1993b: 6–8) and figures therein; for the detailed descriptions of *A.confusum* and *A.seticella*, see He and van Achterberg (1994: 160–163 and of *A.nigrum* Ku and Park, see Ku and Park (1997: 212–213), and figures therein. For the key in this paper, we used the comparative characters of *A.philippinense* and *A.seticella* of specimens collected in Vietnam.

In Vietnam, *Aulacocentrumphilippinense* (Ashmead) was previously reported by Long and Belokobylskij (2003) as solitary parasitoid of the rice leaf folder, *Cnaphalocrocismedinalis* (Pyralidae). All the species found in Vietnam have morphological characters that fit well to *Aulacocentrum* in the key to genera and the diagnosis of this genus provided by van Achterberg (1993b), except *A.glabrum* sp. nov.: marginal cell distinctly narrower medially than basally and more or less broadly widened apically; first metasomal tergite elongate, flat basally or with medio-basal depression, 3–6× as long as its apical width; antenna of both sexes often bicoloured (a variable character in *Aulacocentrum*).

### ﻿Descriptions of species

#### ﻿ Aulacocentrum assitum

Taxon classificationAnimaliaHymenopteraBraconidae

Long & Pham sp. nov.

3F29539B-EB73-54FF-9523-F91A2469793D

https://zoobank.org/91B36A32-E69B-427B-9B30-715174C971E2

Figs 1 , 2 , 16

##### Material.

***Holotype***, ♀, “Macr.**147**” (IEBR), NE Vietnam: Ha Giang, Vi Xuyen, Phong Quang, forest, 22°54'00"N, 104°54'56"E; 650 m a.s.l.; light trap, 26.v.2022, PT Nhi, PV Phu.

##### Description.

**Figure 1.**
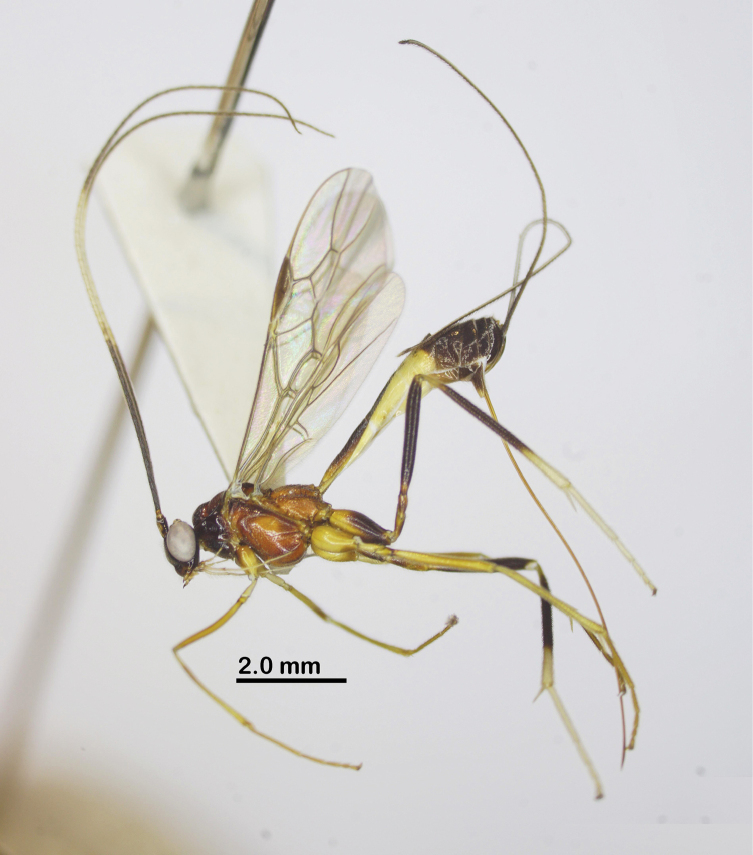
Habitus of *Aulacocentrumassitum* Long & Pham, sp. nov., holotype, female, lateral, “Macr.**147**” (IEBR).

Holotype, female, body length 7.4 mm, fore wing length 6.2 mm, antenna 12.2 mm, ovipositor sheath 7.6 mm (Fig. 1).

**Figure 2.**
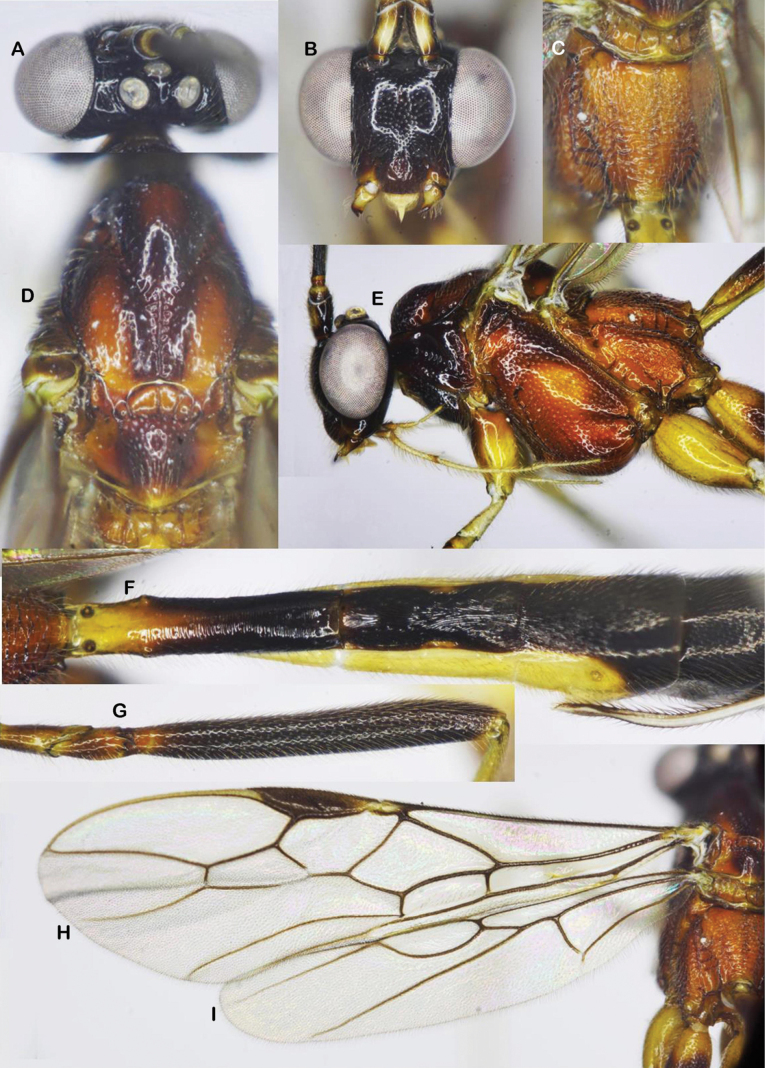
*Aulacocentrumassitum* Long & Pham, sp. nov., holotype, female, “Macr.**147**” (IEBR) **A** head, dorsal **B** head, frontal **C** propodeum, dorsal **D** mesonotum **E** mesopleuron **F** first–third metasomal tergites **G** hind femur **H** fore wing **I** hind wing.

***Head*.** Antenna with 48 flagellomeres; flagellum densely setose; first flagellomere 1.4× second one; length of first and second flagellomeres 7.5 and 5.5× their widths, respectively; subapical antennomere 2.0× its width; apical flagellomere with long spine; in frontal view, width of face 1.2× its length (Fig. 2B); length of maxillary palp 2.2× height of head; face shiny, punctate; malar space as long as basal width of mandible; clypeus convex, finely punctate (Fig. 2B); distance between tentorial pits 1.8× distance from pit to eye margin; in lateral view, eye 3.5× temple medially; head transverse, in dorsal view, head 2.1× as wide as long; and width of head 0.6× median length; temple short, eye 13.5× as long as temple; ocelli large, OOL: OD: POL = 6: 8: 7 (Fig. 2A); frons, temple, and vertex coriaceous.

***Mesosoma.*** Length of mesosoma 1.4× its height (Fig. 2E); pronotal trough crenulate medially, mostly smooth dorsally, sparsely punctate ventrally; propleuron densely punctate (Fig. 2E); middle lobe of mesoscutum without impressions anteriorly; notauli narrow, mostly smooth anteriorly, punctate posteriorly; notauli fused posteriorly, separating by mid-longitudinal rugosity (Fig. 2D); median lobe of mesoscutum smooth, with sparse fine punctures; lateral lobes of mesoscutum mostly smooth, with sparse punctures near notauli; scutellum convex, with dense fine punctures; mesopleuron densely punctate (Fig. 2E); metapleuron rugose-punctate; propodeum foveolate-rugose (Fig. 2C).

***Wings*.** Length of fore wing 2.9× its maximum width (Fig. 2H); length of pterostigma 4.0× its width; fore wing vein SR1 2.3× as long as vein 3-SR; vein r originating behind middle of pterostigma, r: 3-SR: SR1 = 11: 25: 57; cu-a slightly inclivous (Fig. 2H), cu-a: 1-CU1: 2-CU1 = 9: 4: 33; 2-SR: 3-SR: r-m = 17: 25: 7; second submarginal cell of fore wing narrowed distally; hind wing vein cu-a: 1-M: 1r-m = 15: 12: 13; vein 2-SC+R vertical; vein SR strongly bent basally, at constriction mostly touching the frontal wing margin (Fig. 2I).

***Legs*.** Hind coxa sparsely setose; length of femur, tibia, and basitarsus of hind leg 7.8, 17.8, and 14.0× their maximum widths, respectively; left hind trochantellus with two teeth apically (Fig. 2G); tibial spurs straight, setose, length of hind inner and outer tibial spurs 0.4× and 0.3× hind basitarsus combined; length of hind basitarsus 0.4× hind tibia and 1.1× second–fifth tarsal segments.

***Metasoma*.** Length of metasoma 1.5× head and mesosoma combined; first tergite parallel-sided, flat medio-basally, length 5.1× its apical width; mostly smooth anterior of spiracle, transversely rugose from spiracle to apex; median length of second tergite 1.2× third tergite; second tergite narrowed medio-laterally (Fig. 2F), with fine convergent striae; third tergite with fine parallel striae basally; finely punctate apically; fourth–sixth metasomal tergites punctate, densely setose (Fig. 2F); length of ovipositor sheath 1.2× fore wing.

***Colour*.** Head black; scapus dark brown, cream-white ventrally; pedicel yellow; flagellum brown with flagellomeres 9^th^–18^th^ ivory; mandible yellow; palpi whitish yellow; propleuron brown; middle lobe of mesoscutum and scutellum yellowish brown; lateral lobes of mesoscutum, mesopleuron, metapleuron, and propodeum reddish yellow; fore and middle legs yellow; hind coxa yellow basally, brown apically; hind trochanter and trochantellus infuscate; hind femur dark brown; hind tibia mostly brown, pale yellow basally, cream-white apically; hind tibial and tarsus cream-white; tegula brownish yellow; wing membrane hyaline; veins yellowish brown, except vein 1-R1 and parastigma whitish yellow; pterostigma brown, pale yellow apically; metasoma brown, except first tergite basally, second and third metasomal tergites laterally and ventrally pale yellow; ovipositor sheath brown; ovipositor yellow.

**Male.** Unknown.

##### Biology.

Unknown.

##### Etymology.

From *assitus* (Latin for “near”), because this new species is close to *A.seticella* van Achterberg & He, 1994, from China.

##### Distribution.

NE Vietnam (Ha Giang province) (Fig. 16).

##### Notes.

This new species is close to *A.seticella* van Achterberg & He, but differs from the latter by the following characters: 1) length of maxillary palp 2.2× height of head (1.6–1.7× in *A.seticella*); 2) marginal cell of hind wing as wide basally as apically (Fig. 2I) (strongly widened basally in *A.seticella*, see He and van Achterberg 1994: fig. 18); and 3) second metasomal suture distinct, basal 1/3 of third tergite finely striate (second suture indistinct, more than basal 1/2 of third tergite finely striate in *A.seticella*).

#### ﻿ Aulacocentrum glabrum

Taxon classificationAnimaliaHymenopteraBraconidae

Long sp. nov.

9DF4F5C7-119B-5721-9B8F-0AA70371FBEA

https://zoobank.org/798AD0C1-3AFB-4C60-8329-8DDC4FB2DB5C

Figs 3 , 4 , 16

##### Material.

***Holotype***, ♀, “Macr.**050**” (IEBR), S Vietnam: Dong Nai, Vinh Cuu, Phu Ly, forest, 11°22'52.3”’N, 107°03'43.6"E; 107 m a.s.l., light trap, 03.viii.2008, HV Tru.

##### Description.

**Figure 3.**
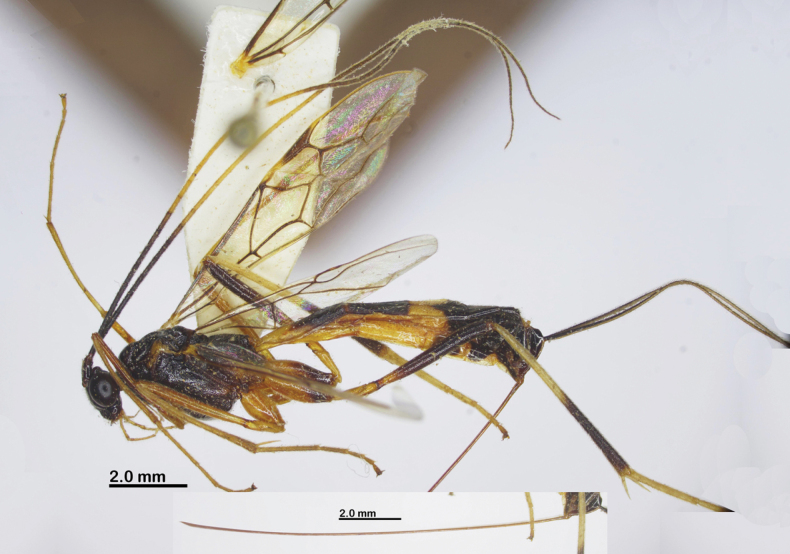
Habitus of *Aulacocentrumglabrum* Long, sp. nov., holotype, female, lateral, “Macr.**050**” (IEBR).

Holotype, female, body length 12.0 mm, fore wing length 9.4 mm, antenna 17.6 mm, ovipositor sheath 13.4 mm (Fig. 3).

**Figure 4.**
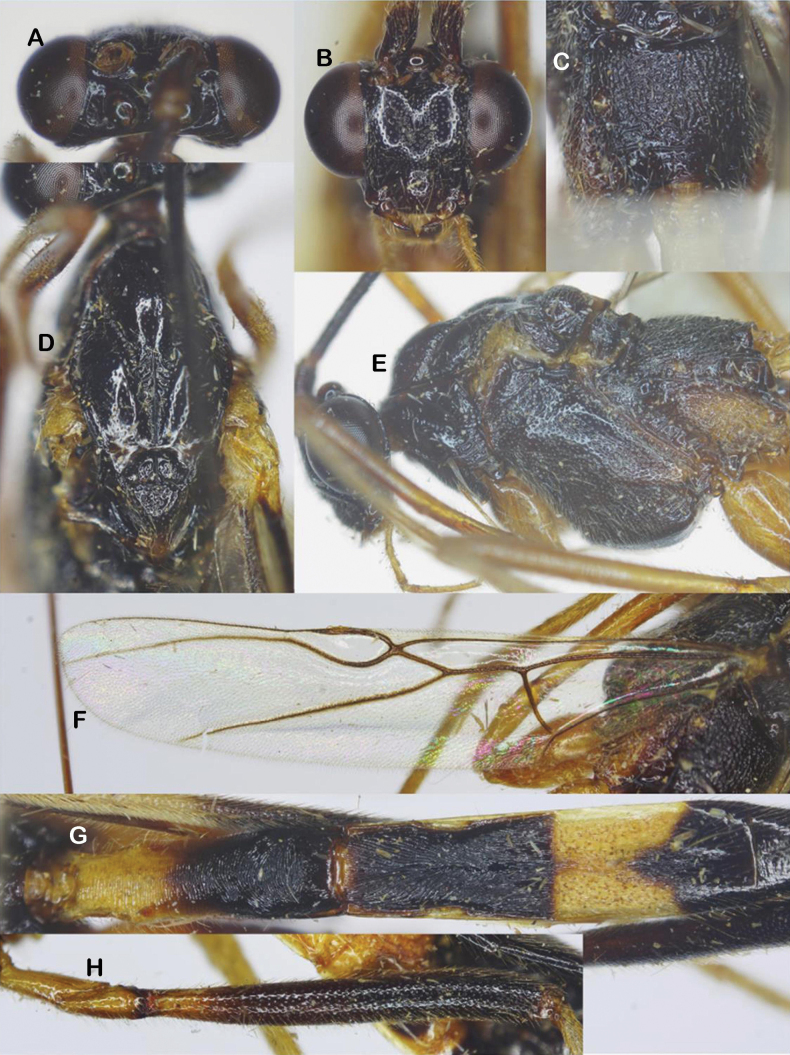
*Aulacocentrumglabrum* Long, sp. nov., holotype, female, “Macr.**050**” (IEBR) **A** head, dorsal **B** head, frontal **C** propodeum **D** mesonotum **E** mesopleuron **F** hind wing **G** first–third metasomal tergites **H** left hind femur, outer side.

***Head*.** Antenna with 51 flagellomeres; first flagellomere 1.3× second one; length of first and second flagellomeres 3.8 and 4.4× their widths, respectively; length of subapical antennomere 2.7× its width; in frontal view, width of face 0.8× its length (Fig. 4B); length of maxillary palp 2.0× height of head; face densely punctate medially, sparsely punctate laterally (Fig. 4B); malar space 0.9× as long as basal width of mandible; clypeus slightly convex ventrally, sparsely finely punctate (Fig. 4B); distance between tentorial pits 1.3× distance from pit to eye margin; in dorsal view, width of head 2.2× median length; temple short, smooth, eye 15.5× temple; ocelli large, OOL: OD: POL = 6: 9: 9 (Fig. 4A); frons smooth.

***Mesosoma.*** Length of mesosoma 1.4× its height (Fig. 4E); pronotal trough finely and densely crenulate medially, shiny smooth dorsally, finely punctate ventrally; propleuron densely punctate (Fig. 4E); middle and lateral lobes of mesoscutum mostly coriaceous, with sparse fine punctures; notauli crenulate anteriorly, strongly converging posteriorly in a sharp V-shaped depression, divided by mid-longitudinal rugosity (Fig. 4D); scutellar sulcus wide, with one median carina, 0.5× as long as scutellum; scutellum convex, rugose-punctate; mesopleuron densely punctate (Fig. 4E); metapleuron rugulose; propodeum reticulate-rugulose (Fig. 4C).

***Wings*.** Length of fore wing 5.2× its maximum width; length of pterostigma 5.2× its width; fore wing vein SR1 2.2× as long as vein 3-SR; r: 3-SR: SR1 = 12: 37: 80; vein 1-CU1 quadrate; cu-a: 2-CU1 = 11: 52; 2-SR: 3-SR: r-m = 21: 37: 10; second submarginal cell of fore wing narrowed distally; hind wing vein 2-SC+R thick, nearly quadrate; vein 1-M weakly curved basally; hind wing vein cu-a: 1-M: 1r-m = 22: 22: 16; vein SR strongly bent basally (Fig. 4F), and marginal cell largely glabrous basally, wider medially than basally and apically, parallel-sided medially and distinctly widened apically (Fig. 4F).

***Legs*.** Hind coxa densely setose latero-ventrally, but without setae dorso-apically; length of femur, tibia, and basitarsus of hind leg 9.1, 17.0, and 12.3× their maximum widths, respectively; left hind trochantellus with four teeth apically (Fig. 4H); length of hind inner and outer tibial spurs 0.4× and 0.3× hind basitarsus, respectively; length of hind basitarsus 0.4× hind tibia and as long as second–fifth tarsal segments combined.

***Metasoma*.** Length of metasoma 1.6× head and mesosoma combined; first tergite deeply concave medio-basally (Fig. 4G), length of first tergite 3.6× its apical width; median length of second tergite 1.1× third tergite; first metasomal tergite transversely striate basally, transverse-obliquely striate medio-subapically, obliquely striate at apex; second tergite with convergent striae on most part of tergite, but with parallel striae apically (Fig. 4G); third tergite with fine striae, mostly smooth apically; remaining metasomal tergites sparsely punctate, with sparse long setae; length of ovipositor sheath 1.4× fore wing.

***Colour*.** Head dark brown; palpi yellow; scapus and pedicellus dark brown; flagellum brown with flagellomeres 8^th^–18^th^ yellow; mesosoma brown; wing veins yellowish brown; tegula yellow; parastigma, pterostigma basally and apically yellow; wing membrane hyaline; fore and middle legs yellow; hind coxa (except apically brown), trochanter and trochantellus yellow; hind tibia brown, except yellow at base; hind tarsus and tibial spurs yellow; metasoma blackish brown, except basal 1/2 of first and third tergites pale yellow; ovipositor sheath brown; ovipositor yellow.

**Male.** Unknown.

##### Biology.

Unknown.

##### Etymology.

From *glaber* (Latin for hairless), referring to the hind wing with both the basal cell apically and the marginal cell basally glabrous.

##### Distribution.

S Vietnam (Dong Nai province) (Fig. 16).

##### Notes.

This new species can be distinguished from other species by the following characters: marginal cell of hind wing largely glabrous basally, and vein 1-CU1 of fore wing quadrate.

#### ﻿ Aulacocentrum imparum

Taxon classificationAnimaliaHymenopteraBraconidae

Long & van Achterberg sp. nov.

A063A767-1FC5-553A-8A43-D0B173104980

https://zoobank.org/3C625000-324F-452E-98A5-3C510B6E3672

**Figure 5.**
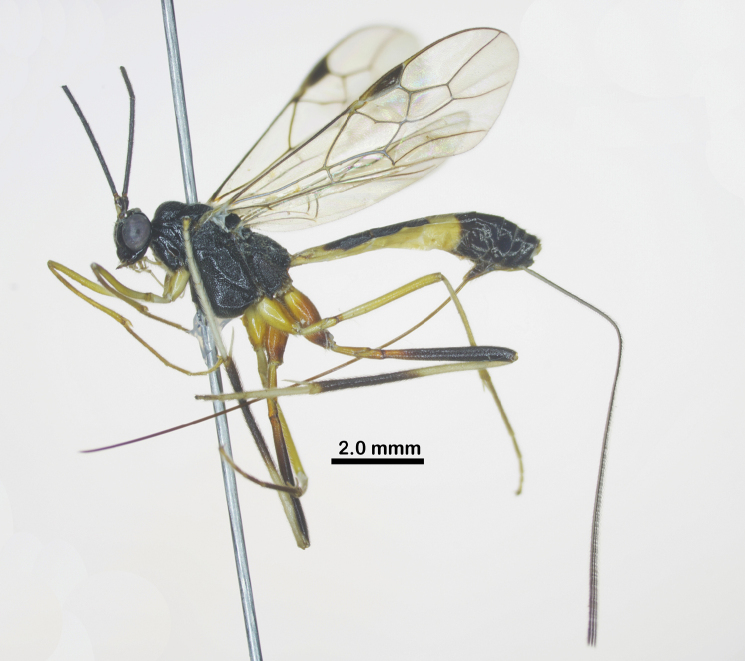
Habitus of *Aulacocentrumimparum* Long & van Achterberg, sp. nov., holotype, female, lateral, “Macr.**172**” (IEBR).

**Figure 6.**
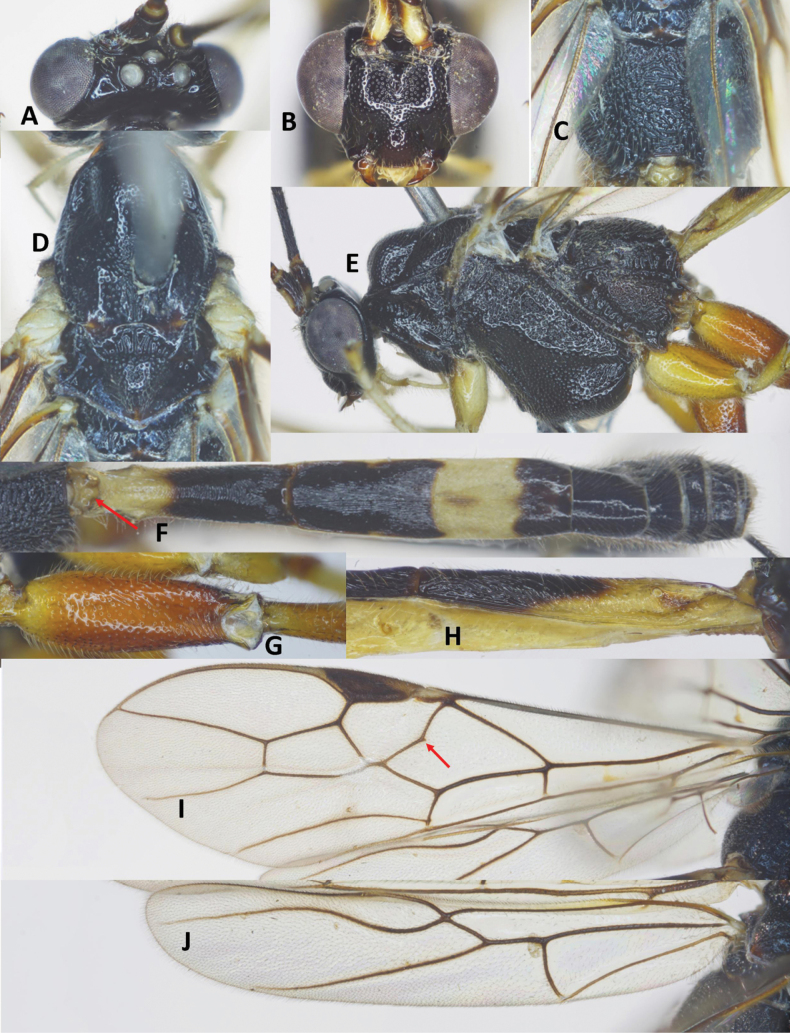
*Aulacocentrumimparum* Long & van Achterberg, sp. nov., holotype, female, “Macr.**172**” (IEBR) **A** head, dorsal **B** head, frontal **C** propodeum **D** mesonotum **E** mesopleuron **F** first-third metasomal tergites, arrow indicates medio-basal depression **G** hind coxa, dorsal **H** first metasomal tergite, lateral **I** fore wing, arrow indicates vein 1-SR+M of fore wing angularly bent medially **J** hind wing.

**Figure 7.**
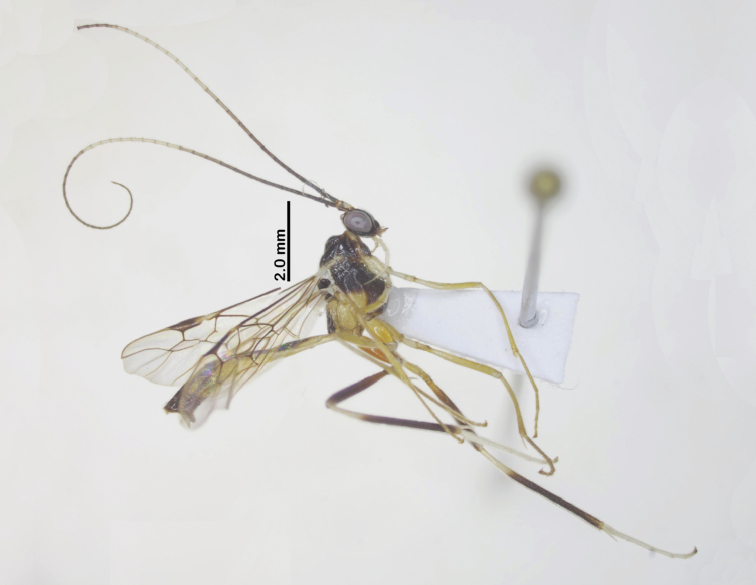
Habitus of *Aulacocentrumimparum* Long & van Achterberg, sp. nov., paratype, male, lateral, “Macr.**170**” (IEBR).

**Figure 8.**
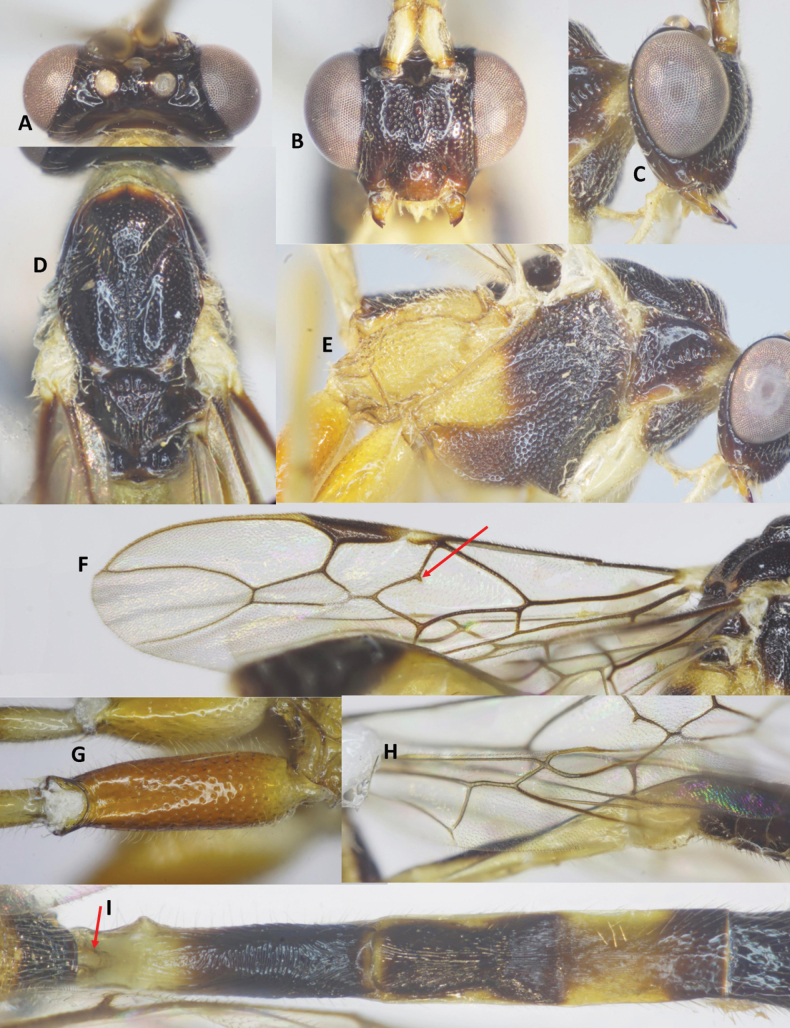
*Aulacocentrumimparum* Long & van Achterberg, sp. nov., paratype, male, “Macr.**170**” (IEBR) **A** head, dorsal **B** head, frontal **C** head, lateral **D** mesonotum **E** mesopleuron **F** fore wing, arrow indicates angular curve of vein 1-SR+M **G** hind coxa, dorsal **H** hind wing (part) **I** first-third metasomal tergites, arrow indicates medio-basal depression.

Figs 5 , 6 , 7 , 8 , 16

##### Material.

***Holotype***, ♀, “Macr.**172**” (IEBR), NE Vietnam: Tuyen Quang, Lam Binh, Thac Nghien, forest, 22°34.334'N, 105°16.762'E; 114 m, light trap, 21.ix.2017, HTHCT. Paratype, 1♂, “Macr.**170**” (IEBR), NE Vietnam: topotypic.

##### Description.

Holotype, female, body length 9.8 mm, fore wing length 7.5 mm, ovipositor sheath 10.5 mm (Fig. 5).

***Head*.** Antenna incomplete, with six flagellomeres remaining; first flagellomere 1.5× second; length of first and second flagellomeres 6.5 and 4.2× their widths, respectively; in frontal view, width of face 0.9× its length (Fig. 6B); length of maxillary palp 1.7× height of head; face densely punctate medially, sparsely punctate laterally (Fig. 6B); malar space 1.1× as long as basal width of mandible; clypeus straight ventrally, sparsely, finely punctate; distance between tentorial pits 1.2× distance from pit to eye margin; in dorsal view, width of head 2.8× median length (Fig. 6A); eye 9.3× as long as temple; ocelli medium-sided, OOL: OD: POL = 9: 9: 10 (Fig. 6A); frons, vertex and temple smooth.

***Mesosoma.*** Length of mesosoma 1.4× its height (Fig. 6E); pronotal trough largely crenulate medially, mostly smooth ventrally and dorsally; propleuron densely punctate (Fig. 6E); middle lobe of mesoscutum rugulose dorsally; punctate ventrally; notauli sparsely crenulate anteriorly, narrowly fused posteriorly with median rugosity (Fig. 6D); scutellar sulcus with five median carina, 0.5× scutellum; scutellum densely punctate; mesopleuron and metapleuron largely rugose-punctate (Fig. 6E); propodeum mostly transversely rugulose medially (Fig. 6C).

***Wings*.** Length of fore wing 3.0× its maximum width (Fig. 6I); length of pterostigma 3.2× its width; vein SR1 of fore wing 2.1× as long as vein 3-SR; r: 3-SR: SR1 = 10: 29: 60; vein 1-SR+M of fore wing angularly bent medially (Fig. 6I); cu-a: 1-CU1: 2-CU1 = 14: 4: 60; 2-SR: 3-SR: r-m = 18: 29: 10; second submarginal cell of fore wing narrowed distally; hind wing with vein 2-SC+R quadrate (Fig. 6J); vein of hind wing cu-a: 1-M: 1r-m = 15: 13: 10; marginal cell sparsely setose, slightly widened apically.

***Legs*.** Hind coxa densely setose latero-ventrally, densely punctate dorsally, with fine oblique striation apically (Fig. 6G); length of femur, tibia, and basitarsus of hind leg 8.4, 16.4, and 8.0× their maximum widths, respectively; left hind trochantellus with three teeth apically; length of hind inner and outer tibial spurs 0.4× and 0.3× hind basitarsus, respectively; length of hind basitarsus 0.3× hind tibia and 1.3× second–fifth tarsal segments combined.

***Metasoma*.** Length of metasoma 1.4× head and mesosoma combined; first tergite slightly concave medio-basally (Fig. 6F), length of first tergite 3.8× its apical width; laterope large (Fig. 6H); second tergite slightly longer than third tergite medially; first metasomal tergite mostly transversely striate; second tergite weakly constricted medio-laterally, with convergent striae on basal 2/3 of tergite, parallel striation on apical third of tergite (Fig. 6F); third tergite finely striate on basal 2/3 of tergite; remaining metasomal tergites coriaceous, with dense setae (Fig. 6F); length of ovipositor 1.4× fore wing.

***Colour*.** Head and mesosoma black; scapus pale yellow, brown dorsally; palpi yellow; fore leg pale yellow, except fore tarsus brownish yellow; middle leg yellow, except middle tarsus brownish yellow; hind coxa brownish yellow, trochanter and trochantellus; hind femur blackish brown, yellow at extreme base; hind tibia blackish brown apically, cream-white basally; hind tibial spurs and tarsus cream-white; tegula cream-white; wing veins brown; parastigma yellow; pterostigma brown, yellow basally and apically; wing membrane hyaline; basal 1/2 of first metasomal tergite pale yellow; apical 1/2 of first tergite and second tergite black; basal 2/3 of third tergite pale yellow, apical 1/3 black; first–third sternites pale yellow and remainder black; ovipositor sheath brown; ovipositor yellow.

##### Variation male.

Paratype, “Macr.**170**” (IEBR), body length 7.5 mm, fore wing length 5.5 mm (Fig. 7).

***Head*.** Antenna with 41 flagellomeres; first flagellomere 1.3× second; length of first and second flagellomeres 6.7 and 5.0× their widths, respectively; in frontal view, width of face 0.9× its length (Fig. 8B); length of maxillary palp 1.4× height of head; face densely punctate medially, sparsely punctate laterally (Fig. 8B); malar space 1.1× as long as basal width of mandible; clypeus weakly convex in lateral view (Fig. 8C); straight ventrally, sparsely punctate; distance between tentorial pits 1.4× distance from pit to eye margin; in dorsal view, width of head 2.7× median length; eye 7.7× as long as temple; OOL: OD: POL = 6: 6: 9 (Fig. 8A); frons, vertex and temple coriaceous.

***Mesosoma.*** Length of mesosoma 1.5× its height; pronotal trough largely crenulate medially, mostly smooth ventrally and dorsally; propleuron rugose-punctate (Fig. 8E); notauli sparsely crenulate anteriorly, narrowly fused posteriorly with median rugosity (Fig. 8D); middle lobe of mesoscutum rugulose dorsally; punctate ventrally; median lobes of mesoscutum sparsely punctate; scutellar sulcus 0.4× scutellum, with one median carina; scutellum densely punctate; mesopleuron rugose-punctate medially, densely punctate ventrally (Fig. 8E); metapleuron rugose-punctate; propodeum mostly transversely rugulose medially.

***Wings*.** Length of fore wing 3.5× its maximum width (Fig. 8F); length of pterostigma 4.8× its width; vein SR1 of fore wing 2.1× as long as vein 3-SR; r: 3-SR: SR1 = 10: 25: 52; vein 1-SR+M of fore wing angularly bent medially (Fig. 8F); cu-a: 1-CU1: 2-CU1 = 6: 3: 37; 2-SR: 3-SR: r-m = 14: 25: 10; second submarginal cell of fore wing narrowed distally; hind wing vein 2-SC+R quadrate (Fig. 8H); vein of hind wing cu-a: 1-M: 1r-m = 16: 12: 10; marginal cell sparsely setose.

***Legs*.** Hind coxa with discrete punctures dorsally, distinctly depressed dorso-apically and mostly smooth (Fig. 8G); length of femur, tibia, and basitarsus of hind leg 8.5, 16.8, and 9.0× their maximum widths, respectively; left hind trochantellus with three teeth apically; length of hind inner and outer tibial spurs 0.4× and 0.3× hind basitarsus, respectively; length of hind basitarsus 0.4× hind tibia and 1.2× second–fifth tarsal segments combined.

***Metasoma*.** Length of metasoma 1.5× head and mesosoma combined; first metasomal tergite parallel-sided, with deep medio-basal depression (Fig. 8I), length 4.0× its apical width; second tergite 1.2× third tergite medially; first metasomal tergite mostly transversely striate; second tergite with convergent striae on basal 2/3 of the tergite, parallel striation on apical 1/3 of tergite (Fig. 8I); third tergite finely striate on basal 2/3 of tergite; remaining metasomal tergites coriaceous, with dense setae.

***Colour*.** Head black; scapus whitish yellow, brown dorsally; palpi whitish yellow; antenna brown basally and apically, with 11^th^–19^th^ flagellomeres ivory; pronotum whitish yellow; mesoscutum blackish brown to black; mesopleuron tricoloured, dark brown dorso-anteriorly, brown ventrally and yellow posteriorly; metapleuron entirely yellow (Fig. 8E); propodeum dark brown posteriorly, yellow anteriorly and laterally; fore and middle legs pale yellow; hind coxa reddish yellow (Fig. 8G); trochanter and trochantellus pale yellow; hind femur dark brown, yellow basally; hind tibia blackish brown in apical 3/4 of the tibia, cream-white in basal 1/4 ; hind tibial spurs and tarsus cream-white; tegula cream-white; wing veins brown; vein 1-R1 and parastigma yellow; pterostigma brown, yellow basally; wing membrane hyaline; basal 1/3 of first metasomal tergite pale yellow; apical 1/2 of first tergite and second tergite blackish brown; basal 2/3 of third tergite yellow, apical 1/3 brown; its remainder black.

##### Biology.

Unknown.

##### Etymology.

From *impar* Latin for unequal, odd, different, because in both sexes, left fore wing with vein 1-SR+M angularly bent medially.

##### Distribution.

NE Vietnam (Tuyen Quang province) (Fig. 16).

##### Notes.

This new species (both sexes) is closely related to *A.philippinense* (Ashmead) but can be separated from the latter by the following characters: 1) first metasomal tergite with basal depression (vs flat in *A.philippinense*); 2) second metasomal tergite (female) weakly constricted medio-laterally (vs distinctly constricted medio-laterally in *A.philippinense*); 3) OOL equal to OD; stemmaticum coriaceous (OOL slightly longer than OD, and stemmaticum rugulose in *A.philippinense*) and 4) vein 1-SR+M of fore wing angularly bent medially (vs evenly curved in *A.philippinense*).

#### ﻿ Aulacocentrum intermedium

Taxon classificationAnimaliaHymenopteraBraconidae

Long & van Achterberg sp. nov.

4544C255-C4B6-5C79-AE50-83CA504E07EA

https://zoobank.org/4800309E-1E86-4407-B01E-39A20A0938EE

Figs 9 , 10 , 16

##### Material.

***Holotype***, ♀, “Macr.**174**” (IEBR), NE Vietnam: Cao Bang, Ha Quang, Yen Son, forest, 22°47'11.2"N, 104°54'06"E, 987 m, light trap, 12.vi.2023, Pham TN, Pham VP, Dang TH.

##### Description.

**Figure 9.**
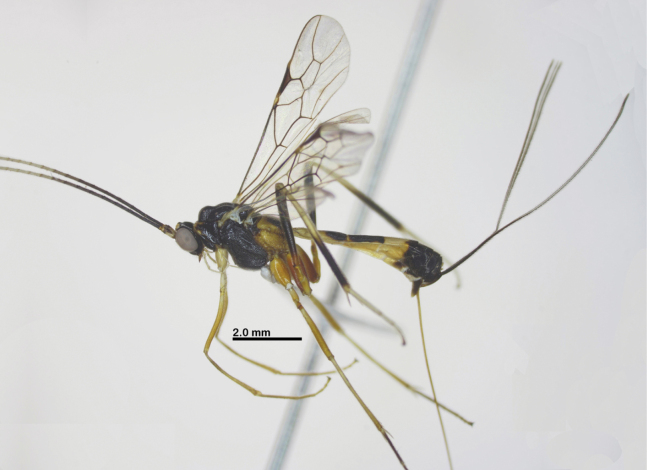
Habitus of *Aulacocentrumintermedium* Long & van Achterberg, sp. nov., holotype, female, lateral, “Macr.**174**” (IEBR).

Holotype, female, body length 8.9 mm; fore wing length 7.8 mm; ovipositor 9.0 mm (Fig. 9).

**Figure 10.**
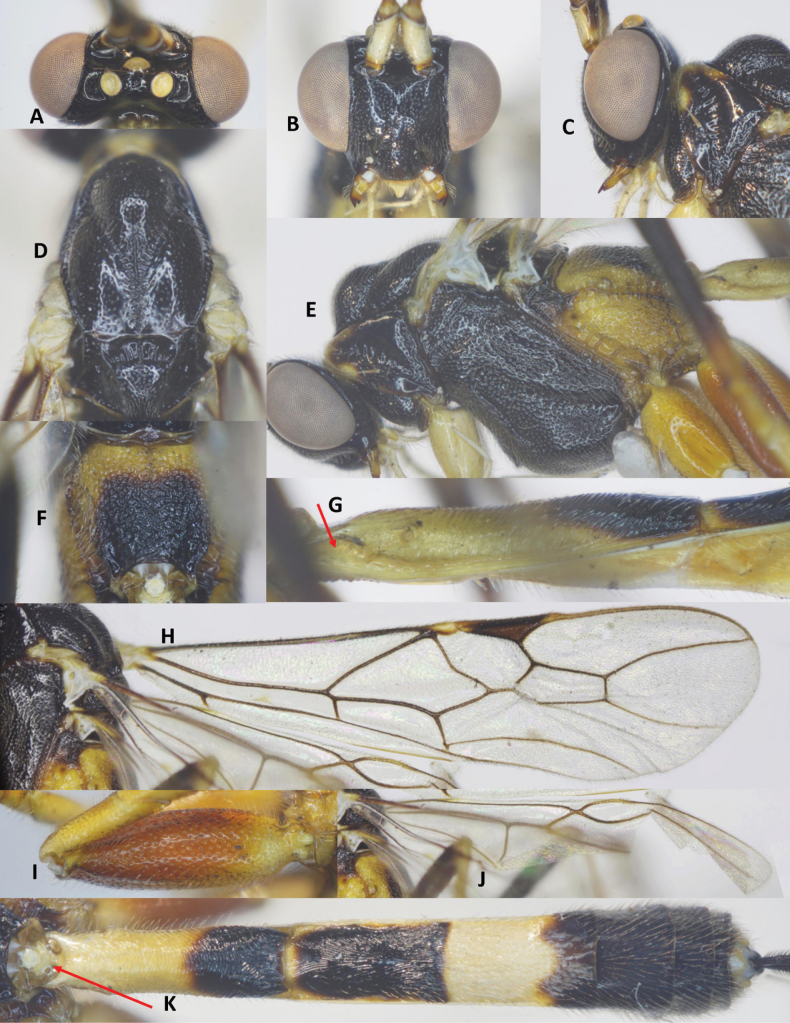
*Aulacocentrumintermedium* Long & van Achterberg, sp. nov., holotype, female, “Macr.**174**” (IEBR) **A** head, dorsal **B** head, frontal **C** propodeum, lateral **D** mesonotum **E** mesopleuron **F** propodeum, dorsal **G** first metasomal tergite, lateral, arrow indicates laterope **H** fore wing **I** hind coxa, dorsal **J** hind wing (part) **K** first-third metasomal tergites, dorsal, arrow indicates medio-basal depression.

***Head*.** Antenna with 47 flagellomeres; first flagellomere 1.2× second one; length of first and second flagellomeres 6.0 and 4.8× their widths, respectively; in frontal view, width of face 0.9× its length (Fig. 10B); length of maxillary palp 1.4× height of head; face densely punctate; malar space as long as basal width of mandible (Fig. 10C); clypeus less convex (Fig. 10C); straight ventrally, sparsely, finely punctate; distance between tentorial pits 1.6× distance from pit to eye margin; in dorsal view, width of head 2.6× median length (Fig. 10A); eye 8.5× as long as temple; OOL: OD: POL = 7: 8: 10 (Fig. 10A); frons, vertex and temple shiny, smooth.

***Mesosoma.*** Length of mesosoma 1.5× its height (Fig. 10E); pronotal trough largely crenulate medially, mostly smooth ventrally and dorsally; propleuron with fine dense punctures (Fig. 10E); middle lobe of mesoscutum densely punctate; lateral lobes of mesoscutum sparsely punctate; notauli narrow, sparsely crenulate anteriorly, narrowly fused posteriorly with median rugosity (Fig. 10D); scutellar sulcus with three median carina, 0.5× scutellum; scutellum densely punctate; mesopleuron and metapleuron largely rugose-punctate (Fig. 10E); propodeum mostly irregularly rugulose (Fig. 10F).

***Wings*.** Length of fore wing 4.1× its maximum width (Fig. 10H); length of pterostigma 4.9× its width; fore wing vein SR1 2.2× as long as vein 3-SR; r: 3-SR: SR1 = 10: 28: 63; cu-a: 1-CU1: 2-CU1 = 4: 9: 50; 2-SR: 3-SR: r-m = 13: 28: 9; second submarginal cell of fore wing narrowed distally; hind wing with vein 2-SC+R horizontal (= longitudinal); vein SC+R1 evenly curved (Fig. 10J); cu-a: 1-M: 1r-m = 10: 15: 15; marginal cell sparsely setose.

***Legs*.** Hind coxa densely setose latero-ventrally, rugose-punctate dorsally, without striation apically (Fig. 10I); length of femur, tibia, and basitarsus of hind leg 9.8, 17.7, and 8.0× their maximum widths, respectively; left hind trochantellus with four teeth in one row; length of hind inner and outer tibial spurs 0.4× and 0.3× hind basitarsus, respectively; length of hind basitarsus 0.3× hind tibia and 1.1× second–fifth tarsal segments combined.

***Metasoma*.** Length of metasoma 1.3× head and mesosoma combined; laterope large, fused into a groove posteriorly (Fig. 10G); first tergite evenly widened apically, with medio-basal depression (Fig. 10K); length of first tergite 4.1× its apical width (Fig. 10K); first metasomal tergite parallel-sided, with curved striation medially; second tergite 1.1× third tergite medially (Fig. 10K); second tergite weakly constricted medially, with convergent striae on basal 2/3 of tergite, with parallel striation apically; third tergite mostly coriaceous, with superficial micro-striae basally; remaining metasomal tergites coriaceous, with dense setae.

***Colour*.** Scapus largely whitish yellow, pale brown dorso-apically; flagellum dark brown, with 9^th^–18^th^ middle flagellomeres ivory; palpi cream-white; fore leg yellow except coxa, trochanter and trochantellus whitish yellow; middle leg yellow, except basitarsus and second brown; hind coxa reddish yellow, hind trochanter and trochantellus yellow, hind femur blackish brown to black, reddish yellow at extreme base; apical 1/2 of hind tibia blackish brown, pale yellow basally; hind tibial spurs and tarsus cream-white; pronotum yellow; mesonotum black; metapleuron yellow; propodeum black medio-posteriorly, yellow basally and ventrally; tegula whitish yellow; wing veins brown; parastigma yellow; pterostigma dark brown, yellow basally and apically; wing membrane hyaline; basal 2/3 of first metasomal tergite pale yellow, apical 1/3 black; second tergite entirely black; basal 2/3 of third tergite whitish yellow, black apically; the remainder blackish brown to black; hypopygium brown; ovipositor sheath brown; ovipositor yellow.

**Male.** Unknown.

##### Biology.

Unknown.

##### Etymology.

From *inter* in Latin meaning “between”, because this new species is intermediate between *A.imparum* sp. nov. and *A.philippinense*.

##### Distribution.

NE Vietnam (Cao Bang) (Fig. 16).

##### Notes.

This new species is similar to *A.philippinense* (Ashmead) but can be separated from the latter by the following characters: 1) first metasomal tergite with basal depression (vs flat in *A.philippinense*); 2) second metasomal tergite weakly constricted medio-laterally (vs distinctly constricted medio-laterally in *A.philippinense*); 3) stemmaticum coriaceous (vs stemmaticum rugulose in *A.philippinense*); and 4) hind coxa rugose-punctate dorsally with oblique striation dorso-laterally (vs nearly smooth with transverse striation dorso-apically in *A.philippinense*).

#### ﻿ Aulacocentrum simulatum

Taxon classificationAnimaliaHymenopteraBraconidae

Long sp. nov.

3EB76FE4-1FAA-5177-9474-21AD56992A5B

https://zoobank.org/EE45A15F-983B-4EA8-A99B-5B8D9B2C2AA2

Figs 11 , 12 , 16

##### Material.

***Holotype***, ♀, “Macr.**011**” (IEBR), NC Vietnam: Thua Thien-Hue, A Luoi, A Roang forest, 16°06'36.0"N, 107°24'30.7"E, 700 m, light trap, 7.vi.2006, HV Tru.

##### Description.

**Figure 11.**
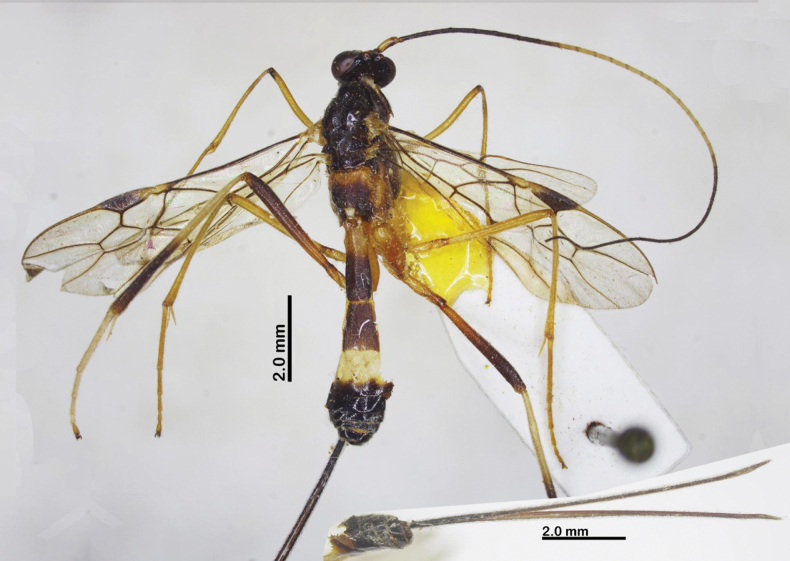
Habitus of *Aulacocentrumsimulatum* Long, sp. nov., holotype, female, dorsal, “Macr.**011**” (IEBR).

Holotype, female, body length 10.3 mm, fore wing length 7.7 mm, antenna 15.0 mm, ovipositor 8.3 mm (Fig. 11).

**Figure 12.**
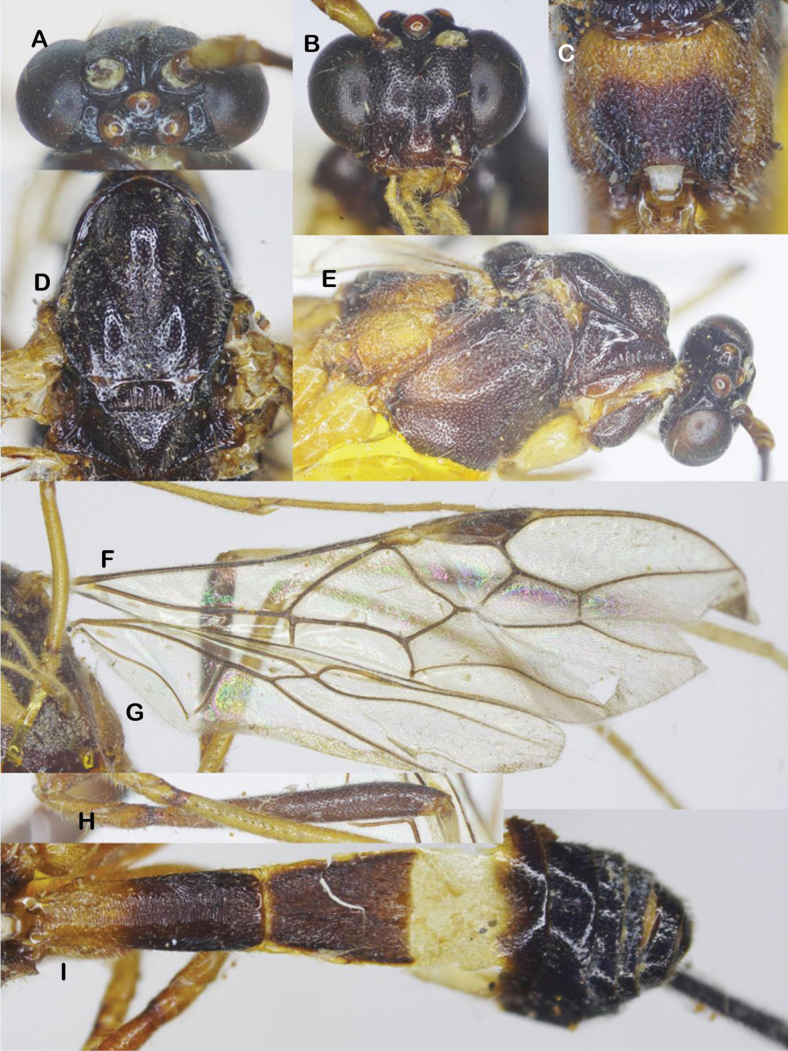
*Aulacocentrumsimulatum* Long, sp. nov., holotype, female, “Macr.**011**” (IEBR) **A** head, dorsal **B** head, frontal **C** propodeum, dorsal **D** mesonotum **E** mesopleuron **F** fore wing **G** hind wing **H** hind trochanter, trochantellus and femur **I** metasoma.

***Head*.** Antenna incomplete, with 44 flagellomeres remaining, with 13 middle flagellomeres ivory; first flagellomere 1.3× second one; length of first and second flagellomeres 6.7 and 5.0× their widths, respectively; length of subapical antennomere 2.7× its width; in frontal view, width of face 0.9× its length (Fig. 12B); length of maxillary palp 1.5× height of head; face plough, with rather dense punctures; malar space 0.8× as long as basal width of mandible; clypeus with fine sparse punctures (Fig. 12B); distance between tentorial pits 2.0× distance from pit to eye margin; in lateral view, eye 5.3× temple; in dorsal view, head transverse, 2.3× as wide as long dorsally (Fig. 12A); eye 7.0× as long as temple; ocelli large, OOL: OD: POL = 4: 8: 8 (Fig. 12A); frons smooth, with median groove; stemmaticum rugose-punctate; vertex and temple punctate.

***Mesosoma.*** Length of mesosoma 1.4× its height (Fig. 12E); pronotal trough largely crenulate medially, punctate ventrally, smooth medio-dorsally; propleuron rugose-punctate (Fig. 12E); middle lobe of mesoscutum mostly rugose-punctate; lateral lobes densely punctate; notauli sparsely punctate anteriorly, converging posteriorly in a V-shaped depression, transverse rugose area separated by median carina-like rugosity (Fig. 12D); scutellar sulcus deep, with six carinae, 0.5× as long as scutellum; scutellum densely punctate; mesopleuron and metapleuron largely rugose; propodeum rugose-punctate basally and ventrally; coarsely rugulose medio-posteriorly (Fig. 12C).

***Wings*.** Length of fore wing 3.2× its maximum width (Fig. 12F); pterostigma rather broad, length of pterostigma 3.5× as long as width medially; fore wing vein SR1 2.5× as long as vein 3-SR; r: 3-SR: SR1 = 10: 25: 63; vein cu-a vertical; cu-a: 1-CU1: 2-CU1 = 10: 3: 46 (Fig. 12F); vein r-m oblique; 2-SR: 3-SR: r-m = 17: 25: 9; second submarginal cell of fore wing narrowed apically; hind wing with vein 2-SC+R horizontal (= longitudinal) (Fig. 12G); vein SC+R1 distinctly bent (Fig. 12G); cu-a: 1-M: 1r-m = 25: 22: 15; marginal cell of hind wing weakly widened basally, slightly narrowed medially, nearly parallel-sided posteriorly (Fig. 12G).

***Legs*.** Length of femur, tibia, and basitarsus of hind leg 8.3, 14.3, and 7.7× their maximum widths, respectively; left hind trochantellus with seven teeth in two rows apically (Fig. 12H); length of hind inner and outer tibial spurs 0.3 and 0.2× hind basitarsus, respectively; length of hind basitarsus 0.4× hind tibia and 1.2× second–fifth tarsal segments combined.

***Metasoma*.** Length of metasoma 1.3× head and mesosoma combined; first tergite deeply concave medio-basally (Fig. 12I), length of first tergite 3.1× its apical width; median length of second tergite 1.1× third tergite; first metasomal tergite with transverse striation on most of tergite, irregular striation at apex (Fig. 12I); second tergite with convergent striation on basal 2/3 of tergite, parallel striation on apical 1/3 of tergite; third tergite with fine parallel striation on basal 4/5 of tergite, apical 1/5 of tergite smooth and sparsely setose; remaining metasomal tergites smooth with sparse setae; length of ovipositor 1.1× fore wing.

***Colour*.** Head blackish brown; scapus nearly pale yellow entirely, except outer side brownish yellow; flagellum brown basally and apically, 8^th^–19^th^ middle flagellomeres ivory; palpi yellow; mesonotum dark brown to black; metapleuron, propodeum basally yellow, dark brown apically; fore and middle legs yellow; hind leg yellow, except hind femur apically, hind tibia apically brown; tegula yellow; wing veins brown; wing membrane yellow, parastigma and pterostigma basally yellow; ovipositor sheath brown; ovipositor yellow.

**Male.** Unknown.

##### Biology.

Unknown.

##### Etymology.

From *simulo* (Latin for “imitate, copy”), because this new species is similar to *A.glabrum* sp. nov.

##### Distribution.

NC Vietnam (Thua Thien-Hue province) (Fig. 16).

##### Notes.

The new species is closely related to *A.glabrum* sp. nov., but differs from the latter by the following characters: 1) hind wing vein cu-a distinctly longer vein 1-M (25: 22) (vein cu-a as long as 1-M in *A.glabrum*); 2) left hind trochantellus with six teeth (four teeth in *A.glabrum*); 3) marginal cell of hind wing sparsely setose (largely glabrous in *A.glabrum*); and 4) first metasomal tergite transversely striate (partly obliquely striate in *A.glabrum*).

###### Newly recorded species

#### ﻿ Aulacocentrum seticella

Taxon classificationAnimaliaHymenopteraBraconidae

van Achterberg & He, 1994

4737FB52-9228-558A-AD3C-536FC1D6DB0F

Figs 13 , 16

Aulacocentrum seticella van Achterberg & He, 1994: 160, figs 2–23, 40.

##### Material examined.

♀, “Bracn.**056**” (IEBR), NE Vietnam: Tuyen Quang, Na Hang, Thanh Tuong, forest, 22°19′01.0″N, 105°24′02″E, 162 m, MT, 15.x.2016, KD Long.

##### Diagnostic characters.

**Figure 13.**
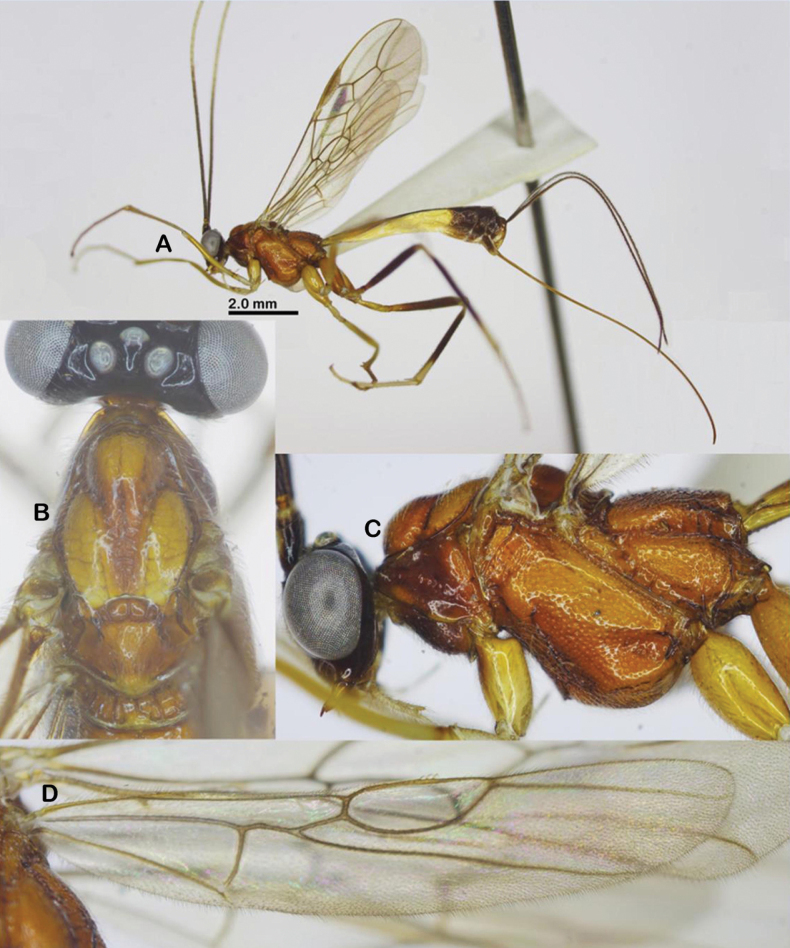
*Aulacocentrumseticella* van Achterberg & He, 1994, “Macr.**056**” (IEBR) **A** habitus, female, lateral **B** mesonotum **C** mesopleuron **D** hind wing.

Based on specimen collected in Vietnam, female, body length 7.5 mm; antenna with 50 antennomeres, with nine middle flagellomeres ivory; fore wing 6.0 mm; ovipositor 7.9 mm (Fig. 13A); length of maxillary palp 1.6× height of head; ratio of length of fore wing veins r: 3-SR: SR1 = 10: 23: 41, and 2-SR: 3-SR: r-m = 13: 23: 6; notauli converging posteriorly in distinct V-shaped depression (Fig. 13B); mesopleuron widely depressed medio-posteriorly (Fig. 13C); hind wing with vein 2-SC+R vertical; vein 1-M curved basally; vein SR mostly touching anterior margin of the wing (Fig. 13D); metasoma 1.5× as long as head and mesosoma combined; first metasomal tergite nearly parallel-sided; length of first tergite 5.2× its apical width; second tergite distinctly constricted medially, with convergent striation; more than basal 1/2 of third tergite finely, longitudinally striate; the remainder coriaceous, densely setose; ovipositor 1.7× metasoma in lateral view, and 1.3× as long as fore wing.

**Male.** Unknown.

##### Biology.

Unknown.

##### Distribution.

Eastern Palaearctic: Japan; Korea; Oriental: China (Guangxi, Guizhou); India, Indonesia (Java, Sumatra), Malaysia (Sabah), Singapore, NE Vietnam (Tuyen Quang province: Na Hang NP) (Fig. 16).

###### Previously recorded species

#### ﻿ Aulacocentrum philippinense

Taxon classificationAnimaliaHymenopteraBraconidae

(Ashmead, 1904)

2801621E-B099-5727-B3D5-28D0459E1E12

Figs 14 , 15 , 16

Macrocentrus philippinensis Ashmead, 1904: 145.

##### Material examined.

**1**♀, “Macr.**088**” (IEBR), S Vietnam: Dong Nai, Vinh Cuu, Phu Ly, TWC, forest, 11°22.612'N, 107°03.594'E, 82 m, light trap, 7.vi.2020, PT Nhi; 2♀, “Macr.**112**”, “Macr.**113**” (IEBR), NE Vietnam: Ha Giang, Bac Me, Minh Ngoc, forest, 22°43'47.2”’N, 105°12'21.3"E; 207 m, light trap, 21.vii.2019, DT Hoa; 1♀, “Macr.**168**” (IEBR), NW Vietnam: Son La, Thuan Chau, Chieng Bom, forest, 21°21'11"N, 103°36'24"E, 1100 m, light trap, 01.v.2016, HV Tru; 1♀, “Macr.**169**” (IEBR), NE Vietnam: Tuyen Quang, Na Hang, Trung Phin, forest, 22°30'13.68"N, 105°23'23.82"E, light trap, 18.ix.2017, HTHCT; 1♀, “Macr.**175**” (IEBR), NE Vietnam: Ha Giang, Dong Van, Pho Bang, light trap, 12.vi–17.vi.2023, Dinh Dieu Thuy; 1♂, “Macr.**126**” (IEBR), S Vietnam: Dong Nai, Vinh Cuu, Phu Ly, Suoi Rong, forest, 11°29'10.3"N, 107°09'58.8"E, 285 m, light trap, 12.vii.2020, PV Phu.

##### Description.

**Figure 14.**
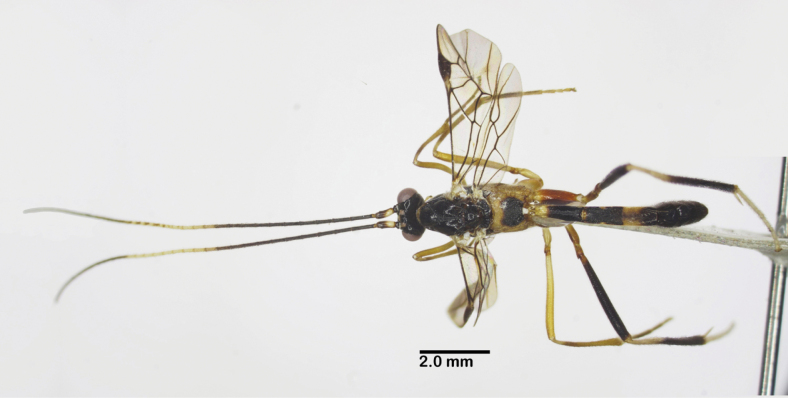
Habitus of *Aulacocentrumphilippinense* (Ashmead), male, dorsal, “Macr.**126**” (IEBR).

Male, “Macr.**126**” (IEBR), body length 8.4 mm, fore wing length 5.9 mm (Fig. 14).

**Figure 15.**
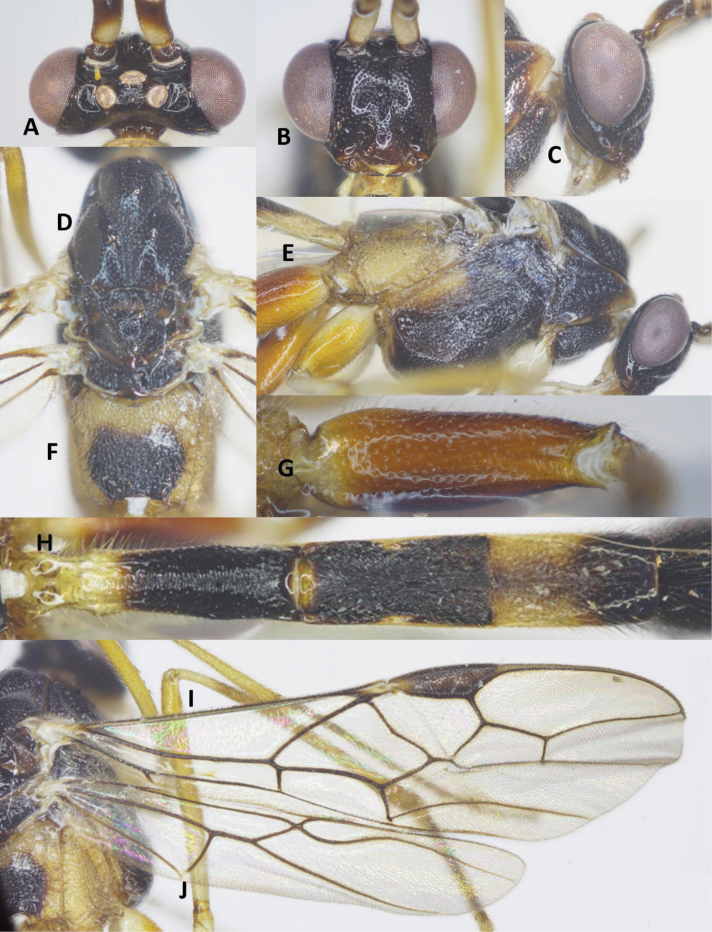
*Aulacocentrumphilippinense* (Ashmead), male, “Macr.**126**” (IEBR) **A** head, dorsal **B** head, frontal **C** head, lateral **D** mesonotum **E** mesopleuron **F** propodeum, dorsal **G** hind coxa **H** first-third metasomal tergites, dorsal **I** fore wing **J** hind wing.

***Head*.** Antenna incomplete, with 31 flagellomeres remaining; first flagellomere 1.4× second one; length of first and second flagellomeres 6.8 and 5.0× their widths, respectively; in frontal view, width of face 0.9× its length (Fig. 15B); length of maxillary palp 1.7× height of head; face densely punctate medially, sparsely punctate laterally; malar space as long as basal width of mandible; clypeus straight ventrally, sparsely, finely punctate; distance between tentorial pits 1.5× distance from pit to eye margin; in dorsal view, width of head 2.4× median length (Fig. 15A); eye 8.3× as long as temple; ocelli medium-sided, OOL: OD: POL = 8: 8: 10 (Fig. 15A); frons, vertex, and temple shiny, smooth; stemmaticum rugose-punctate.

***Mesosoma.*** Length of mesosoma 1.3× its height (Fig. 15E); pronotal trough largely crenulate medially, mostly smooth ventrally and dorsally; propleuron densely punctate (Fig. 15E); middle lobe of mesoscutum rugulose dorsally; punctate ventrally; notauli sparsely crenulate anteriorly, narrowly fused posteriorly with median rugosity (Fig. 15D); scutellar sulcus 2.6× scutellum; scutellum densely punctate; mesopleuron and metapleuron largely rugose-punctate (Fig. 15E); propodeum mostly transversely rugulose (Fig. 15F).

***Wings*.** Length of fore wing 3.3× its maximum width (Fig. 15I); length of pterostigma 3.6× its width; fore wing vein SR1 2.1× as long as vein 3-SR; r: 3-SR: SR1 = 10: 25: 53; cu-a inclivous (Fig. 15I), cu-a: 1-CU1: 2-CU1 = 11: 5: 47; 2-SR: 3-SR: r-m = 16: 25: 8; second submarginal cell of fore wing narrowed distally; hind wing with vein 2-SC+R horizontal (= longitudinal); vein 1-M weakly curved basally (Fig. 15J); cu-a: 1-M: 1r-m = 15: 10: 10; marginal cell sparsely setose.

***Legs*.** Hind coxa elongate, densely setose latero-ventrally, but without setae dorso-apically; length of femur, tibia, and basitarsus of hind leg 8.2, 14.4, and 8.7× their maximum widths, respectively; left hind trochantellus with four teeth in one row (Fig. 15G); length of hind inner and outer tibial spurs 0.6× and 0.3× hind basitarsus, respectively; length of hind basitarsus 0.4× hind tibia and 1.1× second–fifth tarsal segments combined.

***Metasoma*.** Length of metasoma 1.4× head and mesosoma combined; first tergite nearly flat medio-basally (Fig. 15H); length of first tergite 3.8× its apical width; second tergite 1.1× third tergite medially (Fig. 15H); first metasomal tergite mostly transversely striate; second tergite with convergent striae on basal 2/3 of the tergite, parallel striation on 1/3 apical of tergite; third tergite finely striate on 2/3 basal of tergite; remaining metasomal tergites coriaceous, with long setae.

***Colour*.** Pale-yellow; scapus largely whitish yellow, brown dorsally and outer side laterally; flagellum brown, with 11^th^–19^th^ middle flagellomeres cream-white; palpi cream-white; fore leg pale yellow except coxa, trochanter and trochantellus cream-white; middle leg yellow, except coxa basally, trochanter and trochantellus paler; hind coxa yellow, hind trochanter and trochantellus dirty yellow, hind femur entirely, apical 2/3 of hind tibia blackish brown; 1/3 of hind tibia and tarsus cream-white to whitish yellow; pronotum brown, whitish yellow dorsally; mesonotum blackish brown to black; propodeum black, pale yellow basally and ventrally; metapleuron whitish yellow; wing veins brown; tegula whitish yellow; wing membrane hyaline; basal 1/3 of first metasomal tergite pale yellow, apical 2/3, and second tergite entirely black; third tergite whitish yellow basally, blackish brown apically; remainder black; ovipositor sheath brown; ovipositor yellow.

##### Distribution.

**Figure 16.**
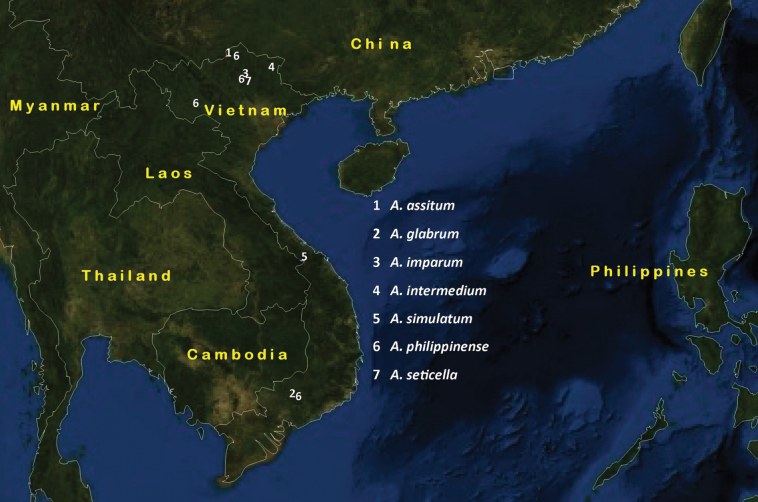
Distribution map of *Aulacocentrum* species.

NE Vietnam (Ha Giang, Tuyen Quang); NW Vietnam (Son La); S Vietnam (Dong Nai) (Fig. 16).

## Supplementary Material

XML Treatment for Aulacocentrum

XML Treatment for Aulacocentrum assitum

XML Treatment for Aulacocentrum glabrum

XML Treatment for Aulacocentrum imparum

XML Treatment for Aulacocentrum intermedium

XML Treatment for Aulacocentrum simulatum

XML Treatment for Aulacocentrum seticella

XML Treatment for Aulacocentrum philippinense

## ﻿Additional information

### Author ORCIDs

Nhi Thi Pham https://orcid.org/0000-0001-9304-9863

Khuat Dang Long https://orcid.org/0000-0002-9237-7344

Cornelis van Achterberg https://orcid.org/0000-0002-6495-4853

Cao Thi Quynh Nga https://orcid.org/0000-0001-9369-6649

Pham Van Phu https://orcid.org/0009-0006-0108-5976

Dang Thi Hoa https://orcid.org/0000-0002-8312-2825

### Data availability

All of the data that support the findings of this study are available in the main text.

## References

[B1] Ashmead WH (1904) Descriptions of new genera and species of Hymenoptera from the Philippine Islands. Proceedings of the United States National Museum 28(1387): 127–158. 10.5479/si.00963801.28-1387.127

[B2] Harris RA (1979) A glossary of surface sculpturing. Occasional Papers in Entomology, California Department of Food and Agriculture 28: 1–33. https://antcat.s3.amazonaws.com/1631/Harris_1979_Occasional_Papers_in_Entomology_A_glossary_of_surface_sculpturing.pdf?

[B3] He J van Achterberg C (1994) A revision of the genus *Aulacocentrum* Brues (Hymenoptera: Braconidae: Macrocentrinae) from China. Zoologische Verhandelingen Leiden 68(15): 159–171. https://repository.naturalis.nl/pub/318760/ZM1994068015.pdf

[B4] Ku DS Park JS (1997) A taxonomic study of the genus *Aulacocentrum* Brues (Hymenoptera, Braconidae, Macrocentrinae) from Korea. Korean Journal of Systematic Zoology 13(3): 211–220. https://koreascience.kr/article/JAKO199711920828729.page

[B5] Long KD Belokobylskij SA (2003) A preliminary list of the Braconidae (Hymenoptera) of Vietnam. Russian Entomological Journal 12(4): 385–398. https://www.elibrary.ru/item.asp?id=9170418

[B6] Sharma V (1978) Taxonomic studies on Indian Braconidae (Hymenoptera). Oriental Insects 12(1): 123–132. https://www.tandfonline.com/doi/abs/10.1080/00305316.1978.10434561

[B7] van Achterberg C (1993a) Illustrated key to the subfamilies of the Braconidae (Hymenoptera, Ichneumonoidea). Zoologische Verhandelingen Leiden 283: 1–189.

[B8] van Achterberg C (1993b) Revision of the subfamily Macrocentrinae Foerster (Hymenoptera, Braconidae) from the Palaearctic region. Zoologische Verhandelingen Leiden 286: 1–110.

[B9] Yu DS van Achterberg C Horstmann K (2016) World Ichneumonoidea 2015. Taxonomy, biology, Morphology and Distribution. Nepean, Ottawa. [database on flash-drive]

